# Single-Cell and Spatial Multi-Omics Analysis Reveal That Targeting JAG1 in Epithelial Cells Reduces Periodontal Inflammation and Alveolar Bone Loss

**DOI:** 10.3390/ijms252413255

**Published:** 2024-12-10

**Authors:** Shuhong Kuang, Jiayu Yang, Zongshan Shen, Juan Xia, Zhengmei Lin

**Affiliations:** Hospital of Stomatology, Guangdong Provincial Key Laboratory of Stomatology, Guanghua School of Stomatology, Sun Yat-sen University, Guangzhou 510000, China; kuangshh3@mail2.sysu.edu.cn (S.K.); yangjy53@mail2.sysu.edu.cn (J.Y.); shenzsh@mail2.sysu.edu.cn (Z.S.)

**Keywords:** periodontitis, macrophage, epithelial cell, Multi-Omics Analysis, cellular communication

## Abstract

Mucosal immunity plays a critical role in the pathogenesis of inflammatory immune diseases. This study leverages single-cell RNA sequencing, spatial transcriptomics, and spatial proteomics to compare the cellular mechanisms involved in periodontitis between humans and mice, aiming to develop precise strategies to protect the gingival mucosal barrier. We identified key conserved and divergent features in cellular landscapes and transcriptional profiles across the two species, underscoring the complexity of inflammatory responses and immune dynamics in periodontitis. Additionally, we revealed a novel regulatory mechanism by which epithelial cells modulate macrophage behavior and inflammation through the JAG1–Notch pathway. Validation through animal experiments revealed that JAG1 inhibition reduces inflammation in epithelial cells, mitigating periodontitis. Our findings advance the understanding of periodontal disease pathogenesis and highlight the importance of integrating human and animal model data to develop treatments aligned with human physiology, offering potential therapeutic targets for controlling inflammation and enhancing tissue regeneration.

## 1. Introduction

Periodontitis is a prevalent and severe chronic inflammatory disease that impacts the supporting structures of the teeth, leading to tooth loss if left untreated [1,2]. Despite advancements in clinical treatment options such as scaling, root planing, and surgical interventions, effective treatments targeting the mechanisms underlying periodontitis progression remain elusive [3,4]. A critical aspect of the pathophysiology of periodontitis is the disruption of the gingival mucosal barrier, a primary interface directly exposed to food and external microbes. This barrier is susceptible to damage by mechanical, chemical, thermal, and biological stimuli leading to dysregulated immune responses and persistent inflammation; this damage not only results in alveolar bone destruction but also significantly diminishes patient quality of life and poses risks to their overall health [5,6].

The resident cells within the gingival mucosa play pivotal roles in regulating immune responses, repairing periodontal tissues, and maintaining barrier homeostasis [7]. For example, the epithelial cells contribute to periodontal homeostasis through antigen presentation [8], whereas the immune cells are important in pathogen clearance and cytokine release [9]. Our previous studies revealed the interactions among these resident cells that compromise gingival barrier stability and induce periodontitis, providing a theoretical basis for developing targeted therapeutic interventions. However, research related to periodontal treatment has focused predominantly on animal models, particularly mice [10,11]. Recent studies have highlighted significant functional and immunological differences between the human and the murine mucosal barriers, which limits the translational potential of findings from murine models to precise human therapies [12,13].

Understanding the differences between human and murine gingival mucosal cells is essential for developing targeted treatment strategies. While single-cell RNA sequencing (scRNA-seq) offers a powerful approach to explore interspecies differences with high resolution [14], integrating techniques such as spatial transcriptomics and spatial proteomics enhances our understanding of tissue-specific cellular behaviors [7,15]. The mucosal immune system is pivotal in the development and progression of inflammatory diseases like periodontitis [16,17]. Previous applications of scRNA-seq have successfully characterized the heterogeneity of epithelial and immune cells in conditions such as inflammatory bowel disease [18], revealing critical insights into cell-specific functions and interactions. Furthermore, these advanced methodologies enable detailed investigations of communication networks between immune and epithelial cells, which are vital for regulating immune responses and preserving mucosal barrier integrity during inflammation [19].

This study aimed to utilize scRNA-seq, spatial transcriptomics, and spatial proteomics to compare epithelial and immune cell populations in the human and murine gingival mucosa. Our findings highlight the close communication between epithelial cells and macrophages in periodontitis. Additionally, spatial transcriptomics and proteomics identified key genes and proteins linked to basal layer inflammation under these conditions. Targeting the JAG1–NOTCH2 axis effectively reduced periodontal inflammation and alveolar bone loss. Overall, this research emphasizes the importance of interspecies comparisons for enhancing the precision and efficacy of therapeutic strategies in inflammatory diseases.

## 2. Results

### 2.1. Single-Cell and Spatial Transcriptomic Analysis Reveals Close Communication Between Epithelial Cells and Macrophages in Periodontitis

**Figure 1:**
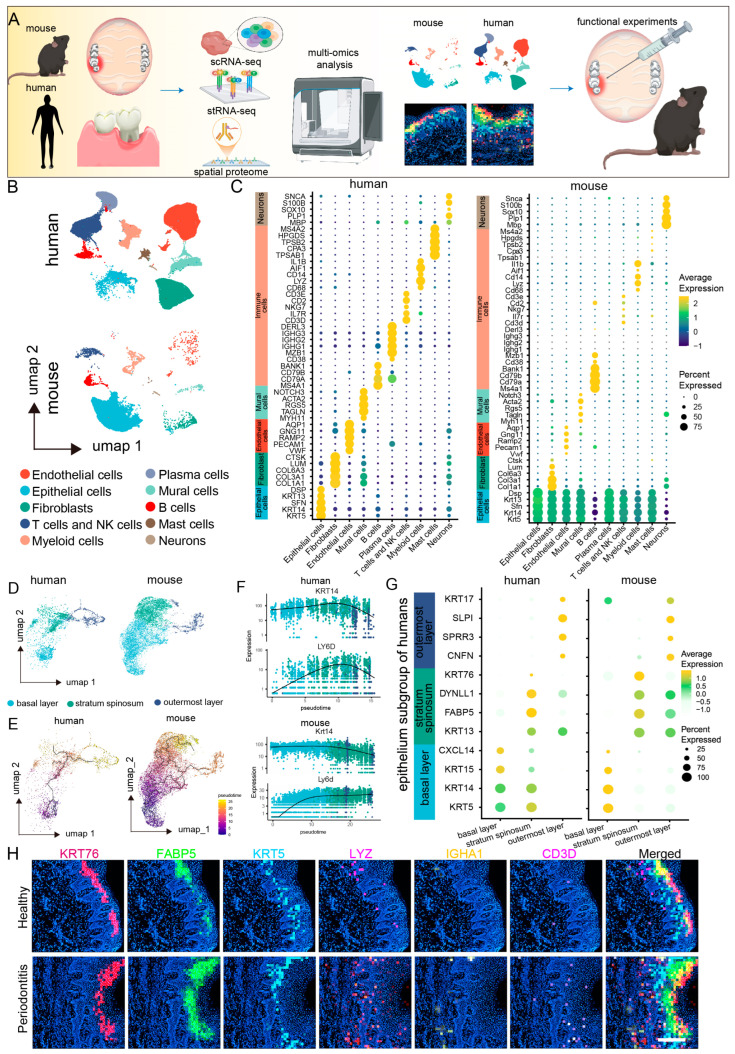
Single-cell and spatial transcriptomic analysis reveals close communication between epithelial cells and macrophages in periodontitis. (**A**). Schematic diagram of the sequencing analysis workflow. (**B**). UMAP plots showing single-cell clustering of the human and mouse gingival mucosa, displaying 10 major cell types: endothelial cells, fibroblasts, epithelial cells, T cells and NK cells, plasma cells, mural cells, myeloid cells, B cells, mast cells, and neurons. (**C**). Dot plots showing gene expression markers from the scRNA-seq data of mice and humans. (**D**). UMAP plots showing clustering of epithelial cell subpopulations in the human and mouse gingival mucosa into 3 subtypes: basal layer, spinous layer, and outer layer. (**E**). Monocle analysis of epithelial cell differentiation trajectories in humans and mice. (**F**). Pseudotime ordering of dynamic gene expression programs in epithelial cells from humans and mice. (**G**). Dot plots showing gene expression markers from the scRNA-seq data of mice and humans. (**H**). Stereo-seq data analysis revealing the relative expression levels of *KRT76*, *FABP5*, *KRT5*, *LYZ*, *IGHA1*, and *CD3D* in the gingiva; scale bar = 200 μm.

To investigate the cellular landscape of mucosal defense responses in periodontitis, we conducted a cross-species single-cell RNA sequencing (scRNA-seq) analysis on mucosal samples from healthy and periodontitis patients, as well as healthy and periodontitis model mice (Figure 1A). After applying quality control metrics and utilizing the Seurat V4 R package, we obtained a total of 69,670 cells from 21 human scRNA-seq datasets, with a median of 24,376 genes per cell, and 24,205 cells from six mouse scRNA-seq datasets, with a median of 21,861 genes per cell (Figure S1A,B). Unsupervised clustering of the scRNA-seq data from both species revealed ten major shared cell types. Uniform manifold approximation and projection (UMAP) clustering highlighted various cell lineages, including endothelial cells, fibroblasts, epithelial cells, T cells, NK cells, plasma cells, pericytes, myeloid cells, B cells, mast cells, and neurons (Figure 1B). Notably, further comparisons of marker gene expression within these cell populations revealed a high degree of similarity between human and mouse gingival mucosal cells, particularly in epithelial cells, myeloid immune cells, and neurons, which presented the highest levels of conserved gene expression across both species (Figure 1C).

Epithelial cells are crucial components of both physical and biological barriers in mucosal defense [20,21]. We extracted and integrated gingival mucosal epithelial cells from both species for clustering analysis. The epithelial cell subpopulation classification was consistent between humans and mice, categorizing the cells into three main subpopulations: basal, spinous, and outer layers (Figure 1D). We observed consistent proportions of these subpopulations, with basal cells being the most abundant, and outer cells the least abundant. However, the proportion of basal cells in mouse gingival epithelial cells was greater than that in human gingival epithelial cells. The distribution patterns of the epithelial cell subpopulations revealed significant differences, particularly in the basal layer, where human cells predominantly clustered in a single basal subgroup (Basal Layer 1), whereas mouse cells clustered in a different subgroup (Basal Layer 2) (Figure S2A,B). Further comparison of marker gene expression between these epithelial cell subpopulations revealed similar expression patterns. In humans, Basal Layer 1 cells predominantly expressed *COL7A1*, *KRT5*, and *KRT14*, whereas Basal Layer 2 cells in mice presented elevated expression of *Txn* and *Krt17*. Interestingly, Basal Layer 2 cells in mice also expressed markers of both Basal Layer 1 and spinous cells, suggesting that Basal Layer 2 cells may represent a transitional state between basal and spinous cells (Figure S2C).

To further explore epithelial cell differentiation trajectories, we performed pseudotime trajectory analysis using Monocle 3. The differentiation trajectory of epithelial cells is largely consistent between species, following a pathway from Basal Layer 1 to Basal Layer 2, then to spinous, and finally to outer layers. However, dynamic gene expression changes during epithelial cell differentiation exhibited species-specific variations (Figure 1E). For example, *KRT14* remained highly expressed in both the basal and the spinous layers of humans and mice, whereas its expression was downregulated in the outer layers. Conversely, *LY6D* expression was increased in human basal cells, maintaining high levels in the spinous and outer layers, but was downregulated in the outer layers of mice (Figure 1F,G).

Spatial transcriptomics revealed the spatial distribution of gene expression and cellular interactions within periodontitis-affected gingiva. Focusing on the epithelial cell subpopulations on the basis of spatial location and cell marker genes, we identified three major subpopulations, namely, the basal, spinous, and outer layers, which was consistent with the scRNA-seq data (Figure 1H). Additionally, we conducted marker analysis for the predominant immune cell types in periodontitis, revealing a significant increase in the number of myeloid immune cells, which were primarily localized in the epithelial basal layer, while no significant changes were observed for B cells or T cells. These findings suggest that myeloid immune cells may interact with basal epithelial cells during periodontitis.

### 2.2. Regulation of Macrophages by Basal Epithelial Cells

**Figure 2:**
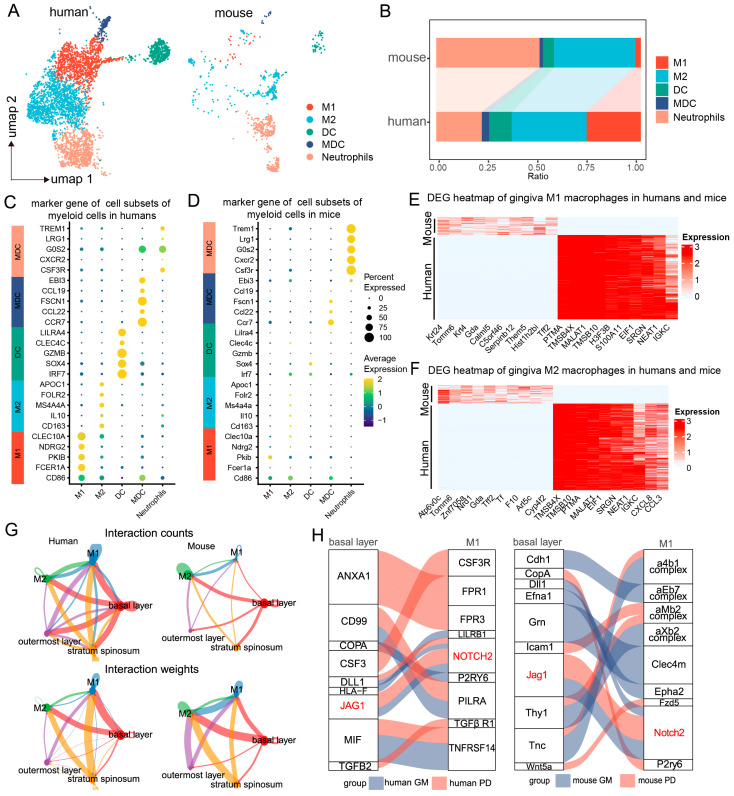
Regulation of macrophages by basal epithelial cells. (**A**). UMAP plots showing clustering of immune cell subpopulations in the human and mouse gingival mucosa, categorized into 5 immune cell subtypes. (**B**). Proportions of each immune cell subtype in humans and mice. (**C**,**D**). Dot plots showing gene expression markers from the scRNA-seq data of mice and humans. (**E**,**F**). Heatmap showing gene set expression for M1/M2 macrophages in the human and mouse gingival mucosa. (**G**). Circle plots showing and comparing alterations in cell–cell communication between epithelial cells and macrophages in the human and mouse gingival mucosa during periodontitis. (**H**). Ligand–receptor interactions between epithelial cells and macrophages in the human and mouse gingival mucosa during periodontitis.

To further explore the role of myeloid immune cells in periodontitis and their interactions with basal epithelial cells, we reclassified the myeloid immune cells using scRNA-seq analysis. We identified six major cell types common to both humans and mice. UMAP clustering highlighted distinct immune lineages, including M1 and M2 macrophages, dendritic cells (DCs), myeloid dendritic cells (MDCs), and neutrophils (Figure 2A). The proportions of M2 cells, DCs, and MDCs in the mouse gingival mucosa were similar to those in the human gingival mucosa, while the M1 cell proportions were significantly greater in the human samples than in the mouse samples, where neutrophils were more abundant (Figure 2B).

Further comparison of cell-specific marker gene expression revealed similarities in the expression profiles between the two species. In humans, M1 macrophages primarily expressed genes such as *CLEC10A*, *NDRG2*, *PKIB*, *FCERIA*, and *CD86*, which were not significantly expressed in the mouse samples, likely due to the lower abundance of M1 cells in the latter (Figure 2C,D). Heatmap analysis revealed that proinflammatory macrophages in mice presented upregulated expression of genes such as *Tomm6*, *Znf705a*, *Mia2*, *Nrd1*, *Tff2*, *Gda*, *Tf*, *Krt24*, and *Arl5c*, whereas human proinflammatory macrophages presented increased expression of *PTMA*, *TMSB4X*, *MALAT1*, *TMSB10*, *H3F3B*, *EIF1*, *S100A11*, *SRGN*, *NEAT1*, and *IGKC* (Figure 2E).

Differential gene expression analysis among these subpopulations provided additional insights. In M2 macrophages, genes such as *Atp6v0c*, *Tomm6*, *Nrd1*, *Znf705a*, *F10*, *Tff2*, *Gda*, *Calml5*, *Tf*, and *Cyp4f2* were upregulated in mice, while *PTMA*, *EIF1*, *SRGN*, *NEAT1*, *IGKC*, *CXCL8*, and *CCL3* were upregulated in humans (Figure 2F). These findings indicate distinct immunoregulatory mechanisms employed by macrophage subtypes during periodontitis, emphasizing both shared and species-specific responses.

By utilizing high-resolution single-cell annotation, we predicted cell communication pairs between epithelial cells and macrophages during periodontitis through the coexpression of ligand–receptor pairs on the basis of interaction structures. Strong interactions were observed between basal epithelial cells and macrophages. Notably, the interaction between human basal epithelial cells and M1 macrophages was stronger than that between mouse basal epithelial cells and M1 macrophages, suggesting interspecies differences in the regulation of macrophages by basal epithelial cells during periodontitis (Figure 2G).

To explore the ligand–receptor interactions between basal epithelial cells and M1 macrophages, we conducted a comparative analysis. The key ligand–receptor pairs in humans included *CD99-PILRA*, *TGFB2-TGFβ* receptor 1, *JAG1-NOTCH2*, *MIF-TNFRSF14*, *CSF3-CSF3R*, and *ANXA1-FPR1*. In mice, the principal ligand–receptor pairs were *GRN-CLEC4M*, *ANXA1-FPR2*, *ICAM1-ITGAL*, *COPA-P2RY6*, and *JAG1-NOTCH2* (Figure 2H). Interestingly, both species exhibited *JAG1–NOTCH2* ligand–receptor interactions, highlighting a common mechanism for epithelial cell–M1 macrophage communication during periodontitis (Figure 2H). These findings suggest that although signaling pathways exhibit species-specific differences, the regulatory interactions between epithelial cells and M1 macrophages are conserved, with *JAG1–NOTCH2* potentially playing a key role.

### 2.3. Analysis of Basal Epithelial Cell Activation Pathways and Downstream Genes During Periodontitis

**Figure 3:**
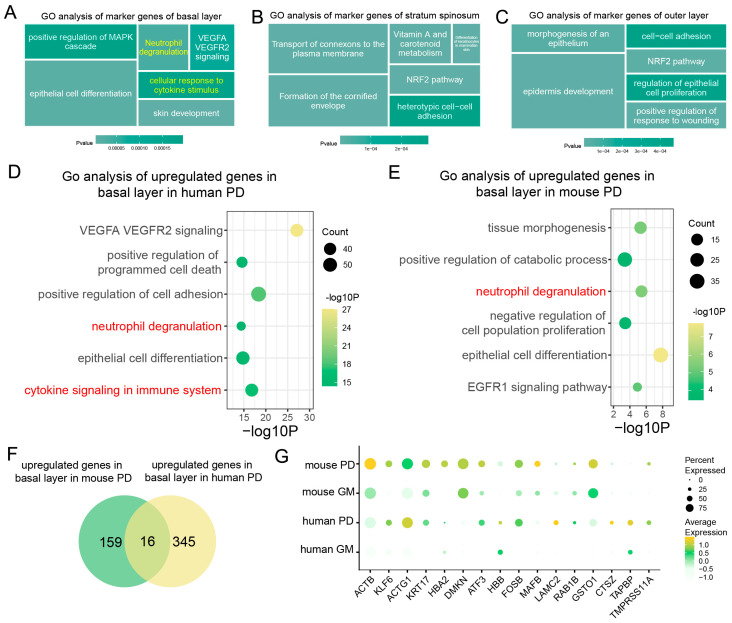
Analysis of basal epithelial cell activation pathways and downstream genes during periodontitis. (**A**–**C**). GO pathway analysis of epithelial cell subtypes in human and mouse gingival mucosa. (**D**,**E**). Dot plots showing gene set expression in the basal epithelial layer of human and mouse gingival mucosa. (**F**). Venn diagrams showing the identification of genes associated with basal epithelial cells in human PD (periodontitis) and mouse PD. (**G**). Dot plots showing gene expression markers from scRNA-seq data in basal epithelial cells in humans and mice with a healthy GM or PD.

To further investigate the roles of epithelial cells during periodontitis, we analyzed gene expression patterns and functional pathways within these cells. Our analysis revealed distinct gene expression patterns and functional roles among the different epithelial cell subpopulations during periodontitis. Specifically, the genes expressed in basal cells were primarily enriched in inflammation-related pathways, including “Positive Regulation of MAPK Cascade”, “Neutrophil Degranulation”, and “Response to Cytokine Stimulus” (Figure 3A). In contrast, the genes expressed in the spinous and outer layers were primarily associated with epithelial proliferation and repair processes (Figure 3B,C).

We focused on comparing the basal epithelial cells of periodontitis-affected humans and mice and analyzed the differentially enriched gene pathways. In both species, these cells were enriched in inflammation-related pathways, indicating a conserved inflammatory response (Figure 3D,E). However, compared with their mouse counterparts, the human gingival epithelial cells demonstrated a unique enrichment in immune cell factor-related pathways (Figure 3D).

In addition to shared inflammatory pathways, species-specific differences in gene expression during periodontitis were also observed (Figure 3F). For example, genes such as *Actb*, *Dmkn*, and *Gsto1* were significantly upregulated in mice, whereas *ACTG1*, *LAMC2*, and *CTSZ* were significantly upregulated in humans (Figure 3G). These findings highlight the crucial role of epithelial cells in immune regulation during periodontitis, particularly concerning pathways related to interactions with immune cells. While both species display similar core inflammatory responses, species-specific mechanisms contribute to the immunoregulatory functions of epithelial cells during periodontitis.

### 2.4. Spatial Proteomics and Spatial Transcriptomics Verify Genes and Proteins Associated with Basal Layer Inflammation in Periodontitis

**Figure 4:**
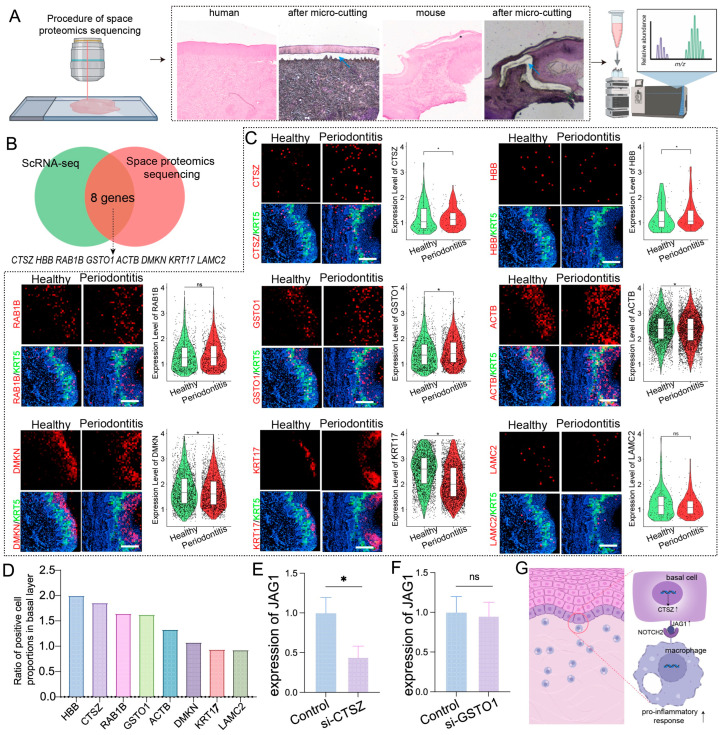
Spatial proteomics and spatial transcriptomics verify genes and proteins associated with basal layer inflammation in periodontitis. (**A**). Schematic diagram of the spatial proteomics workflow; blue arrows indicate the selected area of laser captured. (**B**). Venn diagrams showing the identification of common genes associated with basal epithelial cells via spatial proteomics and scRNA-seq. (**C**). Stereo-seq data analysis revealing the relative expression levels of ACTB, CTSZ, DMKN, GSTO1, HBB, KRT17, LAMC2, and RAB1B in the gingiva; scale bar = 200 μm. (**D**). Proportion of positive cells for each gene in the basal layer of epithelial cells. (**E**,**F**). RT–qPCR analysis of JAG1 expression levels in epithelial cells treated with CTSZ or GSTO1 siRNA. (**G**). Schematic of the mechanism by which epithelial–macrophage interactions cause mucosal inflammation. The data are shown as the means ± SDs of 6 samples per group. Student’s *t*-test, * *p* < 0.05, ns = no significance.

Given that proteins are direct executors of biological functions, revealing their spatial expression is crucial for determining protein localization and function within tissues. We conducted a combined spatial proteomics analysis of basal epithelial cells from humans and mice with periodontitis (Figure 4A). By integrating data from previous scRNA-seq analyses that identified commonly upregulated genes in the basal epithelial cells of both species, we identified eight proteins with high coexpression: ACTB, CTSZ, DMKN, GSTO1, HBB, KRT17, LAMC2, and RAB1B (Figure 4B).

Spatial transcriptomics revealed significant upregulation of ACTB, GSTO1, RAB1B, HBB, DMKN, and CTSZ in basal epithelial cells during periodontitis (Figure 4C,D). Among these genes, CTSZ and GSTO1 presented the highest expression levels associated with inflammation and immune modulation, prompting further investigation into their potential regulation of epithelial JAG1. siRNA-mediated gene silencing experiments demonstrated that CTSZ inhibition significantly reduced JAG1 expression in epithelial cells upon LPS stimulation, whereas no notable changes were observed for GSTO1 (Figure 4E,F). These results suggest that CTSZ may play a key role in the upregulation of JAG1 in epithelial cells during periodontitis, potentially driving a proinflammatory response in macrophages (Figure 4G).

### 2.5. Targeting the JAG1–NOTCH2 Axis Reduces Periodontal Inflammation and Alveolar Bone Loss in Periodontitis

**Figure 5:**
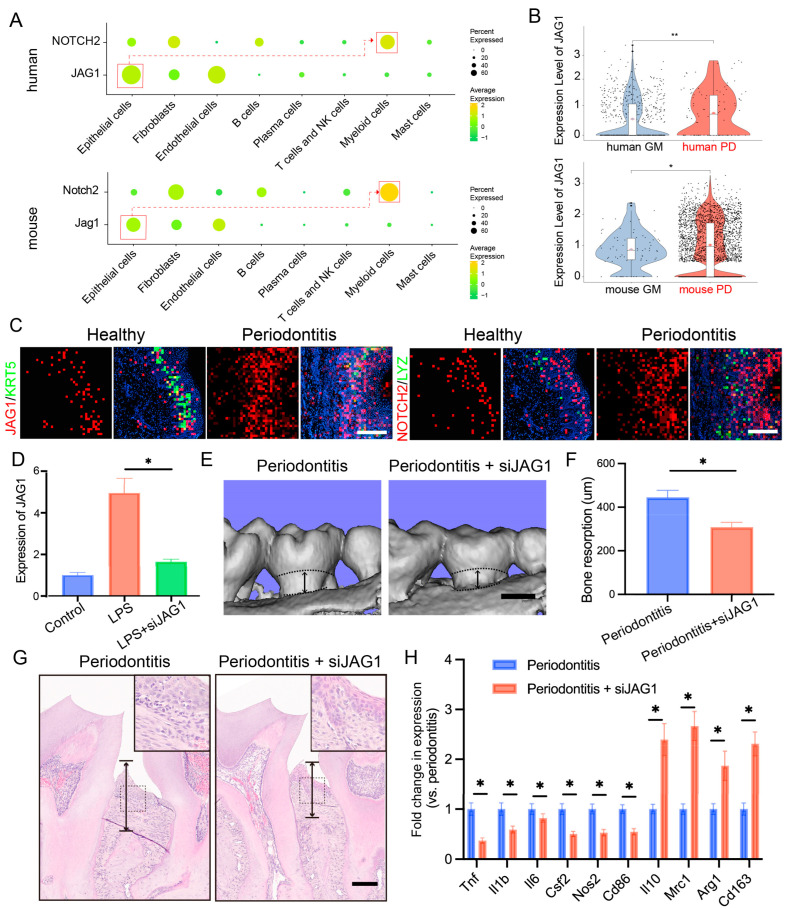
Targeting the JAG1–NOTCH2 axis reduces periodontal inflammation and alveolar bone loss in periodontitis. (**A**). Dot plots showing the expression of JAG1 and NOTCH2 in stromal cells and immune cells. (**B**). Violin plots showing the expression of JAG1 in epithelial cells in the human and mouse gingival mucosa with or without periodontitis. (**C**). Stereo-seq data analysis revealing the relative expression levels of JAG1 and NOTCH2 in the gingiva; scale bar = 200 μm. (**D**). RT–qPCR analysis of JAG1 expression levels in epithelial cells treated with JAG1 siRNA. (**E**,**F**). Three-dimensional reconstructions of the maxilla in each group generated by μCT; the black arrows indicate the distance between cement enamel junction and alveolar bone crest (CEJ-ABC), scale bar = 500 μm. (**G**). Histological HE-stained sections of the periodontium from each group are shown; the black arrows indicate the distance between CEJ-ABC, scale bar = 200 μm. (**H**). RT–qPCR analysis of gene expression in gingival tissues from each group. The data are shown as the means ± SDs of 6 samples per group. Student’s *t*-test, * *p* < 0.05, ** *p* < 0.01.

Considering the critical role of the JAG1–NOTCH2 axis in macrophage-mediated inflammation and our prior findings of a significant upregulation of the JAG1–NOTCH2 ligand–receptor pairs in both human and mouse gingival epithelial cells and macrophages during periodontitis, we investigated the specific role of this axis in epithelial–macrophage interactions. We first measured the expression levels of JAG1 and NOTCH2 across various cell types. JAG1 was primarily expressed in stromal cells, particularly in epithelial cells, whereas NOTCH2 was predominantly expressed in myeloid cells (Figure 5A).

We compared the expression of JAG1 and NOTCH2 under inflammatory and normal conditions and found that JAG1 expression in basal cells and NOTCH2 expression in myeloid cells were significantly elevated under inflammatory conditions (Figure 5B). Spatial transcriptomics further confirmed that JAG1 expression in basal epithelial cells was markedly increased in the context of periodontitis, with concurrent upregulation of NOTCH2 in macrophages (Figure 5C).

To validate the role of the JAG1–NOTCH2 axis in periodontitis, we employed a ligature-induced periodontitis mouse model and administered localized siRNA to suppress JAG1 expression (Figure S3). We then examined the effect of siJAG1 on JAG1 expression at the mRNA level using RT-qPCR. JAG1 expression was reduced after siJAG1 treatment (Figure 5D). IF staining showed that JAG1 expression was significantly reduced in periodontal tissue after siJAG1 treatment (Figure S4A). Micro-CT imaging indicated that the siRNA treatment significantly reduced alveolar bone resorption in the periodontitis model mice (Figure 5E,F). Additionally, hematoxylin and eosin (HE) staining revealed that siJAG1 treatment decreased alveolar bone loss in the affected mice (Figure 5G). Finally, we compared the expression levels of periodontitis-related genes, such as Il1b and Tnf, before and after treatment and found that their expression was notably downregulated (Figure 5H). IF staining showed that TNF-α expression was significantly reduced, while ARG1expression was significantly upregulated in periodontal tissue after siJAG1 treatment (Figure S4B).

These findings indicate that the JAG1–NOTCH2 axis serves as a key mediator of proinflammatory crosstalk between epithelial cells and macrophages during periodontitis, highlighting the potential therapeutic implications of targeting this pathway to reduce inflammation and alveolar bone loss in patients with periodontal disease.

## 3. Discussion

In this study, we integrated scRNA-seq data, spatial transcriptomics, and spatial proteomics from both human and murine models to explore the cellular and molecular mechanisms underlying differences and similarities in the gingival mucosal barrier during periodontitis, supported by in vitro and in vivo experimental validation. We discovered a regulatory mechanism involving the JAG1–Notch signaling axis between basal epithelial cells and M1-type macrophages, which plays a critical role in the progression of periodontitis. A comprehensive analysis of these datasets provided key insights into the conserved and divergent cell populations, gene expression patterns, and cell–cell communication pathways involved in the inflammatory processes of periodontitis. These findings not only deepen our understanding of the pathogenesis of periodontal disease but also offer potential targets for therapeutic interventions aimed at controlling inflammation and promoting tissue repair.

The integration of scRNA-seq data, spatial transcriptomics, and spatial proteomics has facilitated a detailed characterization of cellular populations across species [22,23]. Our study highlights the similarities and critical differences in the cellular and molecular actions within the gingival mucosal barrier in humans and mice during periodontitis. Traditionally, many studies rely solely on mouse models to understand periodontal disease [10,11]; however, our research emphasizes interspecies similarities and crucial discrepancies. For example, we observed conservation in the cell types of the gingival mucosal barrier and molecular functions across the examined species. The composition of barrier cell populations and their expressed markers showed a high degree of similarity between humans and mice, with a similar analysis of epithelial and immune cell subpopulations revealing high congruence.

However, we also identified species-specific differences in the gingival mucosal barrier, particularly in the distribution and activation states of epithelial and immune cell subgroups. Compared with the human samples, the murine model samples exhibited differences in the functions of basal epithelial cells. The analysis of immune cells revealed that the murine model had a more pronounced inflammatory response and greater neutrophil infiltration than the human model did, suggesting that mouse models might overestimate certain inflammatory aspects of the disease. These differences highlight the limitations of solely relying on mouse models and indicate that the results from animal studies may not fully translate to human disease without careful consideration of these discrepancies. By comparing these species, we can refine our preclinical research approaches, ensuring that therapeutic strategies are better tailored to human biology, potentially leading to more precise and effective treatments for periodontal disease.

We also identified a potential mechanism by which epithelial cells regulate macrophages to exacerbate periodontitis. We determined that JAG1 is one of the key molecules by which epithelial cells upregulate the inflammatory state of macrophages. Studies have shown that JAG1 (Jagged1), a ligand in the Notch signaling pathway, can influence cell function and activity through the activation of this pathway [24,25]. Specifically, activation of Notch enhances M1 gene expression and the proinflammatory response in macrophages [26]; macrophage Notch can be activated by LPS-mediated TLR4 stimulation, resulting in the production of proinflammatory cytokines (including TNF, IL-6, IL-10, and IL-12) [27]. However, its role and potential mechanisms of action in periodontitis remain unclear. Therefore, we first clarified through in vitro experiments that epithelial cells can activate the Notch signaling pathway in macrophages via JAG1, promoting their polarization to M1-type macrophages and producing proinflammatory cytokines; the suppression of epithelial cell JAG1 expression through siRNA could eliminate its proinflammatory regulatory effect on macrophages. Furthermore, the use of siJAG1 in a murine periodontitis model significantly reduced the local inflammatory cytokine levels and alveolar bone resorption. These findings indicate that epithelial cells regulate macrophages through the JAG1–Notch axis in both human and murine periodontitis and serve as potential targets for the treatment of periodontitis.

Our approach is novel in that it bridges the gap between purely murine-based research and the need for models more relevant to humans. While previous studies based on animal models have laid the groundwork for understanding the inflammatory processes of periodontitis, the differences we identified suggest that integrating human data early in the treatment development process is necessary. By identifying these interspecies differences, we can move toward more informed strategies to develop treatments that are more likely to succeed in human clinical trials.

Notably, periodontitis is not only a prevalent oral disease but also closely linked to systemic conditions such as metabolic syndrome. Evidence suggests that the chronic inflammatory state associated with periodontitis may exacerbate manifestations of metabolic syndrome, including insulin resistance and dysregulated lipid metabolism [28,29]. Therefore, we propose that targeting the JAG1-Notch pathway could not only reduce the local inflammation associated with periodontitis but also have a positive clinical impact on systemic diseases such as metabolic syndrome.

Our study highlights the role of the JAG1/Notch axis in epithelial cells and M1 macrophages at the gingival mucosa barrier. While the gingival mucosa shares similarities with other mucosal barriers, including immune cell composition and antimicrobial peptide production, it has unique characteristics, such as a propensity for strong inflammatory responses against localized pathogens [30,31]. These differences may limit the generalizability of our findings to other mucosal barriers. However, our cross-species and multi-omics strategy can be extended to other mucosal immunity studies to identify potential therapeutic targets.

While our study reveals the potential role of JAG1 in periodontitis and provides initial validation through a mouse model, we acknowledge several limitations in our current research. First, the ligature-induced periodontitis model used in this study is a classical experimental model that effectively induces localized inflammation and periodontal tissue destruction. But human periodontitis generally follows a chronic course [32,33]. Therefore, the acute nature of this model may not fully replicate the complex pathophysiological processes of human periodontitis. Additionally, the downstream signaling cascade through which epithelial cells regulate macrophage polarization via the JAG1/Notch axis received relatively little attention in our study. Further studies are needed to elucidate how the JAG1/Notch axis regulates macrophage function and contributes to the inflammatory response.

In summary, this study demonstrated the ability to integrate scRNA-seq data from human and murine models to study gingival inflammation. Our results underscore critical immune–epithelial interactions and emphasize the importance of considering species-specific differences. Insights gained from comparing human and mouse data provide a more detailed understanding of periodontal disease and pave the way for developing more precise and effective treatment strategies that account for these interspecies differences. By advancing our understanding of the pathogenesis of periodontal disease, this research lays the foundation for future studies aimed at developing more targeted and human-specific therapeutic approaches for inflammatory periodontal diseases.

## 4. Materials and Methods

### 4.1. Data Acquisition

The scRNA-seq data used in this study were obtained from publicly available datasets deposited in the Gene Expression Omnibus (GEO) database. Specifically, human gingival tissue scRNA-seq data were retrieved from the GSE164241 dataset. Mouse gingival tissue scRNA-seq data were obtained from the GSE228635 and GSE254766 datasets. These datasets were selected on the basis of their comprehensive profiling of immune and epithelial cell populations in gingival tissues, as well as their relevance to the study objectives.

The spatial transcriptomics data used in this study were obtained from the Genome Sequence Archive (GSA) database (https://bigd.big.ac.cn/gsa-human; accessed on 28 September 2024) under the accession code HRA003217.

### 4.2. Patient Recruitment and Tissue Collection

We collected human gingival samples from patients undergoing tooth extraction procedures at the Hospital of Stomatology, Sun Yat-sen University, Guangzhou. Each sample complied with the Chinese GCP, ICH GCP, and relevant regulations and was approved by the Medical Ethics Committee of the Hospital of Stomatology, Sun Yat-sen University (No. KQEC-2022-14-01). Informed consent was obtained from the participants before they were included in our study. Patients with a history of any medical condition other than periodontitis were excluded. The periodontitis samples presented periodontal pockets with depths >4 mm and bleeding on probing. The healthy samples showed no signs of periodontal disease and presented no deep periodontal pockets (depth <3 mm) or bleeding on probing.

### 4.3. Spatial Proteomics of the Human Gingival Samples

The spatial proteomics workflow includes several key steps: (1) tissue sectioning and staining; (2) microdissection of regions of interest; (3) microsample preparation; (4) mass spectrometry; (5) database search; (6) data analysis. The gingival samples were embedded in paraffin and sliced into 5–10 µm sections, which were attached to MMI (MMI GmbH, Eching, Germany) membranes on slides. The slides were subsequently stained with HE to assess tissue morphology and inflammation via laser capture microdissection (LCM, Eching, Germany). For LCM, the slide to be tested was placed on the microscope slide frame, and then the cut sample was placed on the fixed slide. Next, the MMI software (v5.1 # 262; Eching, Germany) was opened, the entire sample was scanned with a 4× mirror, the area to be cut was dragged and selected, and the focal length was adjusted. Subsequently, the system was switched to the 10× magnification area, and the focal length was adjusted again. Then, a mouse or electronic pen was used to circle and select the area to be cut. Finally, the “CUT CELL” button was clicked to cut. After the cutting process was complete, the centrifuge tube covered with an adhesive coating was placed upside down on a glass slide to adhere the cut sample to it. Five microliters of lysis buffer was added to the lid of the EP tube containing the tissue sample, which was subsequently transferred to a new EP tube. Next, a 95 °C heat denaturation treatment was performed in the PCR machine for 10 min. After 10 min of noncontact ultrasonic treatment, the samples were centrifuged at 10,000× *g* for 1 min. The supernatant was diluted with 5 mL of 50 mM TEAB, and 2 µL of 0.5 µg/mL trypsin was added; the reaction was performed at 37 °C overnight in a PCR machine. Finally, an SDB-RPS column was used for the desalination treatment, followed by vacuum drying and mass spectrometry detection. The mass spectrometry data were collected via computer software to obtain the identification of and quantitative information on the peptides and proteins. The databases used in this study were Mus musculus UP000000589 (protein count: 54,822, database: UniProt, loading time: 7 March 2024) and Homo sapiens SP (protein count: 20,434, database: UniProt, download time: 7 March 2024).

### 4.4. Animal Experimentation

Ethical considerations: The use of publicly available datasets in this study complied with the data access and usage policies of the GEO database. All animal experiments were conducted in accordance with protocols approved by the Institutional Animal Care and Use Committee (IACUC) of Sun Yat-sen University (SYSU-IACUC-2024-002851). Eight-week-old male C57BL/6J mice, purchased from the Sun Yet-sen University Animal Supply Center, were used in this study. The mice were housed under specific pathogen-free (SPF) conditions with a 12 h light/dark cycle and provided access to food and water ad libitum.

To induce gingival inflammation, the mice were anesthetized with 4% isoflurane, and a 5–0 silk ligature was tied around the maxillary second molars, with the ligature situated in the gingival sulcus, to induce periodontitis. For the in vivo si-JAG1 treatment, an siJAG1 solution was locally injected into the gingival mucosa after ligature. si-JAG1 (sense: 5′-GUGCCAGUUAGAUGCAAAUTT-3′, antisense: AUUUGCAUCUAACUGGCACTT-3′) was purchased from Kidan Bioscience (Guangzhou, China) and was modified with cholesterol to improve in vivo delivery. The injection solution was prepared with enzyme-free physiological saline, and the injection dose for each animal was 3 nmol. An equal amount of vehicle (enzyme-free physiological saline) was locally injected into the gingival mucosa after ligature placement in the control group. The mice were monitored daily, and after 10 days, they were sacrificed via CO_2_ euthanasia followed by cervical dislocation.

### 4.5. Tissue Collection and Processing for the Animal Study

After euthanasia, the periodontal tissues were harvested. The tissues were immediately placed in ice-cold PBS and processed for histological analysis and gene expression studies. For the histological examination, the tissues were fixed in 4% paraformaldehyde (PFA) for 24 h, embedded in paraffin, and sectioned at a thickness of 5 μm. The sections were stained with HE to assess tissue morphology and inflammation.

For the gene expression analysis, the fresh gingival tissues were snap-frozen in liquid nitrogen and stored at −80 °C until RNA extraction. RNA was extracted using the TRIzol reagent (Thermo Fisher Scientific, Waltham, MA, USA) according to the manufacturer’s protocol. RNA purity was measured using a Nanodrop spectrophotometer, ensuring A260/A280 ratios between 1.8 and 2.0 and A260/A230 ratios above 2.0 for all samples. Reverse transcription was performed using a reverse transcription kit (Takara, Tokyo, Japan). Quantitative real-time PCR (qRT–PCR) was conducted using SYBR Green Master Mix (Thermo Fisher Scientific), and the expression levels of the target genes, including inflammatory cytokines, were normalized to that of the housekeeping gene GAPDH.

### 4.6. Cell Culture

The epithelial cells were cultured in 24-well plates. When the cells reached 50% confluence, they were treated with siRNA (at a concentration of 10 nM). After 48 h, the cells were collected, and total RNA was extracted using TRIzol reagent for quantitative RT–PCR to validate the expression level of JAG1.

### 4.7. uCT Analysis

The maxillae harvested from the mice in the different groups were fixed in 4% PFA overnight. The mandibles were subsequently washed with PBS, dehydrated with 75% ethanol, placed in standardized cylindrical sample holders, and subjected to high-resolution HCT (Scano Medical AG, Bassersdorf, Switzerland). The imaging parameters were as follows: 70 kV, 114 mA, 20 μL increments, and a 3000-millisecond integration time. A three-dimensional image analysis software, Materialise MIMICS (version 20.0; Leuven, Belgium), was used to reconstruct the images and analyze the imaging data.

### 4.8. Data Preprocessing and Quality Control

The scRNA-seq data downloaded from GEO were processed using the Seurat (v5.0) package for alignment and quantification of gene expression. Feature–barcode matrices were generated for both the human and the mouse datasets. Initial quality control (QC) was performed to filter out low-quality cells, which were defined as those with fewer than 500 genes detected or with greater than 15% mitochondrial gene content.

### 4.9. Data Integration and Comparative Analysis

To integrate the human and mouse datasets, we employed the Seurat (v5.0) package. The datasets were normalized using the SCTransform method, and highly variable genes were identified for each dataset. We then used the biomaRt (v 2.60) package and harmony package (v 1.2) to align homologous genes between the two species. After alignment, dimensionality reduction was performed using principal component analysis (PCA), followed by uniform manifold approximation and projection (UMAP) for visualization. Clustering was conducted using the Louvain algorithm, and clusters were annotated on the basis of canonical marker genes for epithelial cells, fibroblasts, and immune cell populations.

To assess the conservation of cell populations between human and mouse gingival tissues, we performed cross-species comparisons via orthologous gene mapping. Differentially expressed genes (DEGs) between inflamed and noninflamed tissues were identified using the Wilcoxon rank-sum test, with adjusted *p* values calculated via the Benjamini–Hochberg correction method.

### 4.10. Cell-Cell Communication Analysis

To infer intercellular communication between immune and epithelial cells in both the human and the mouse datasets, we used the CellChat (v1.6) package. This analysis identified potential ligand–receptor interactions that regulate immune responses and epithelial barrier integrity. The communication networks were visualized using chord diagrams and heatmaps, and pathway enrichment analysis was performed to identify key signaling pathways involved in inflammation and tissue repair.

### 4.11. Immunofluorescence Staining

Gingival sections were deparaffinated and hydrated. After fixation and permeabilization, the sections were incubated with primary antibodies against JAG1, TNF-α, and ARG1, separately at 4 °C overnight, followed by fluorescently labeled secondary antibodies at room temperature for 1 h. DAPI was used to stain the nuclei. Images were captured using a fluorescence microscope (ZEISS Microscopy, Jena, Germany) under identical exposure conditions for all samples. The quantification of mean fluorescence intensity (MFI) was performed using ImageJ software (version 1.52a; NIH, USA).

### 4.12. Statistical Analysis

Statistical analyses were conducted using R (v4.4.1). Data are presented as the means ± standard deviations (SDs) for normally distributed variables or as medians with interquartile ranges (IQRs) for nonnormally distributed variables. The significance of differences between groups was determined using Student’s *t* test or the Mann–Whitney U test, as appropriate. For comparisons involving more than two groups, one-way analysis of variance (ANOVA) followed by Tukey’s post hoc test was used. A *p* value of 0.05 was considered statistically significant.

## Data Availability

The scRNA-seq data and the spatial transcriptomics data are contained within the article and Supplementary Materials; The data presented in this study are available on request from the corresponding author. The spatial proteomics data are not publicly available due to ongoing studies.

## Supplementary Materials

The following supporting information can be downloaded at: https://www.mdpi.com/article/10.3390/ijms252413255/s1.
