# Supplementary material for: Single-Cell and Spatial Multi-Omics Analysis Reveal That Targeting JAG1 in Epithelial Cells Reduces Periodontal Inflammation and Alveolar Bone Loss
Source: Int J Mol Sci. 2024 Dec 10;25(24):13255. doi: 10.3390/ijms252413255 (PMC11675447; doi:10.3390/ijms252413255)
Supplement: Supplementary file 1 [file ijms-25-13255-s001.zip › ijms-3329418-supplementary.pdf]

Article

Single-Cell and Spatial Multi-Omics Analysis Reveal That Targeting JAG1 in Epithelial Cells Reduces Periodontal Inflammation and Alveolar Bone Loss

Shuhong Kuang †, Jiayu Yang †, Zongshan Shen, Juan Xia * and Zhengmei Lin *

Hospital of Stomatology, Guangdong Provincial Key Laboratory of Stomatology, Guanghua School of
Stomatology, Sun Yat-sen University, Guangzhou 510000, China; kuangshh3@mail2.sysu.edu.cn (S.K.); yangjy53@mail2.sysu.edu.cn (J.Y.); shenzsh@mail2.sysu.edu.cn (Z.S.)

* Correspondence: xiajuan@mail.sysu.edu.cn (J.X.); linzhm@mail.sysu.edu.cn (Z.L.)

† These authors contributed equally to this work.

Supplementary Materials

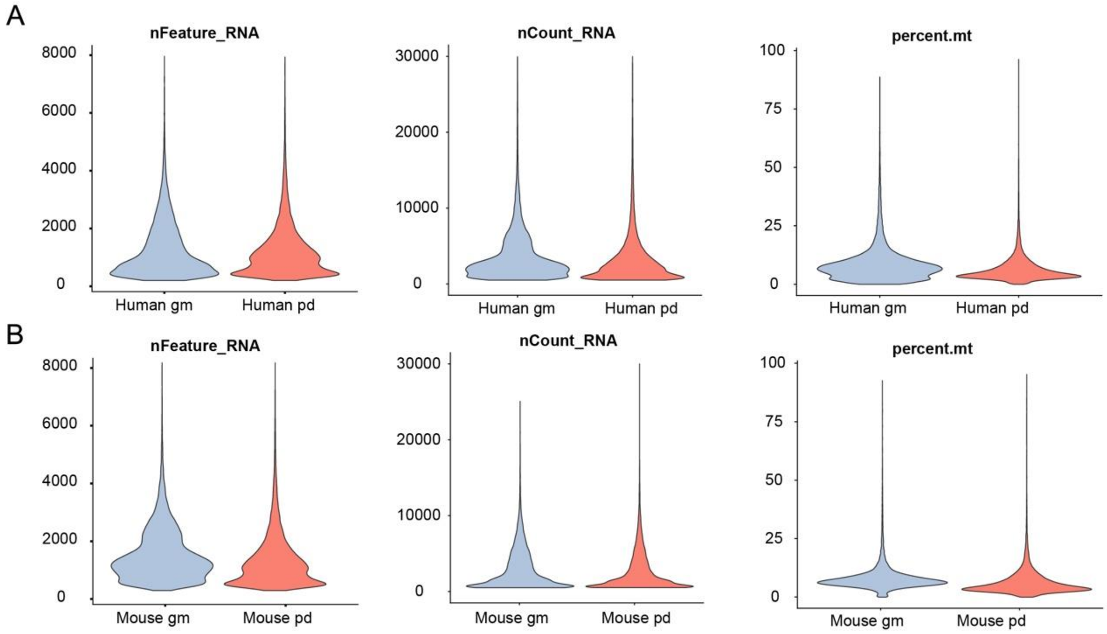

Figure S1. Characterization of single-cell sequencing data from cell samples from humans or mice with or without periodontitis. A. Correlation analysis among nCount RNA, nFeature and percent.mt of cell samples from humans. B. Correlation analysis among nCount RNA, nFeature and percent.mt of cell samples from mice.

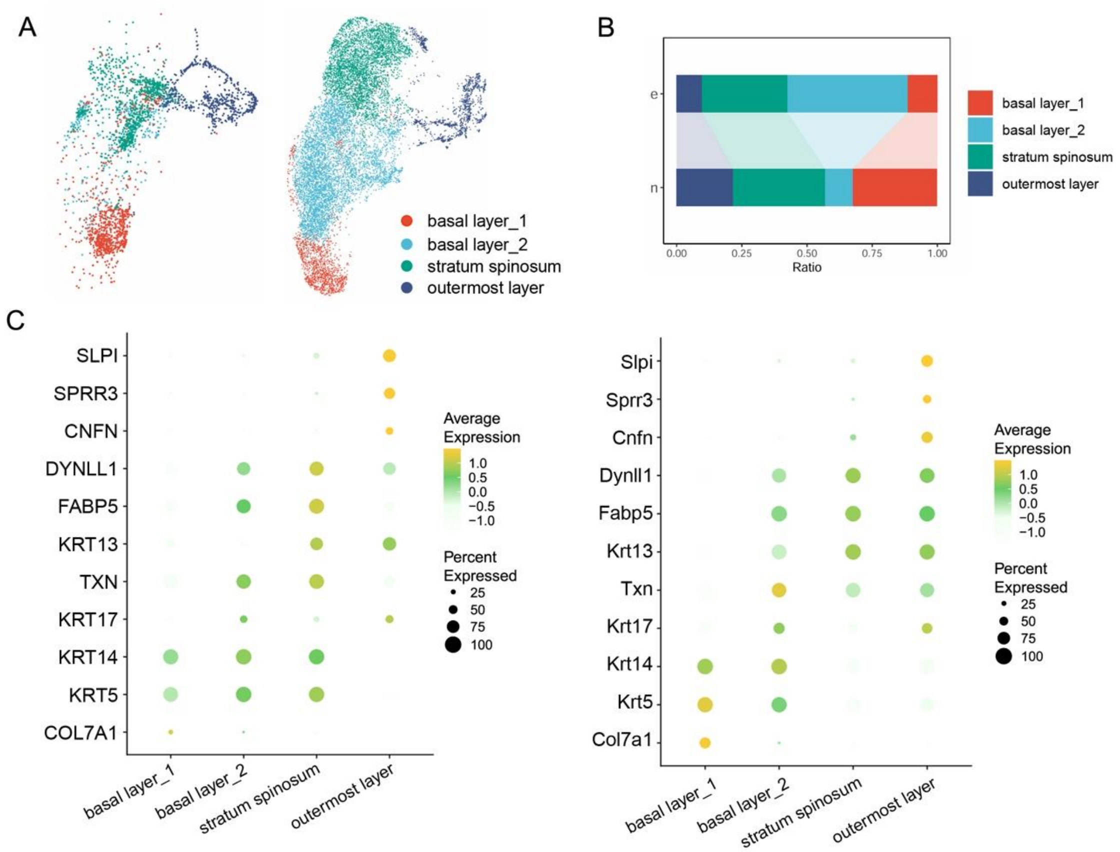

Figure S2. Analysis of epithelial cell types in the gingival mucosa of humans and mice with periodontitis. A. UMAP plots showing clustering of epithelial cell subpopulations in human and mouse gingival mucosa into 4 subtypes: basal layer_1, basal layer_2, the spinous layer, and the outer layer. B. Proportions of each epithelial cell subtype in humans and mice. C. Dot plots showing gene expression markers from the scRNA-seq data of mice and humans.

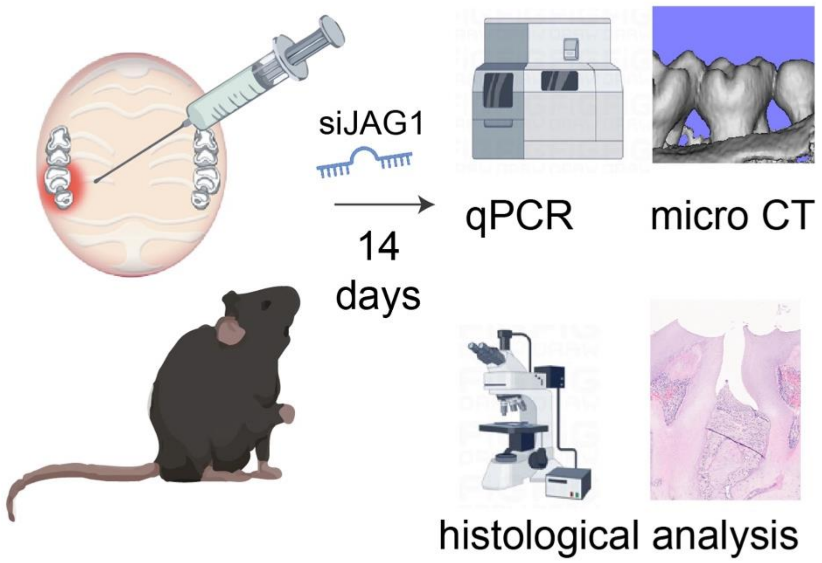

Figure S3. Periodontitis mouse model and administered localized siRNA to suppress JAG1 expression Schematic diagram of the animal experiment workflow.

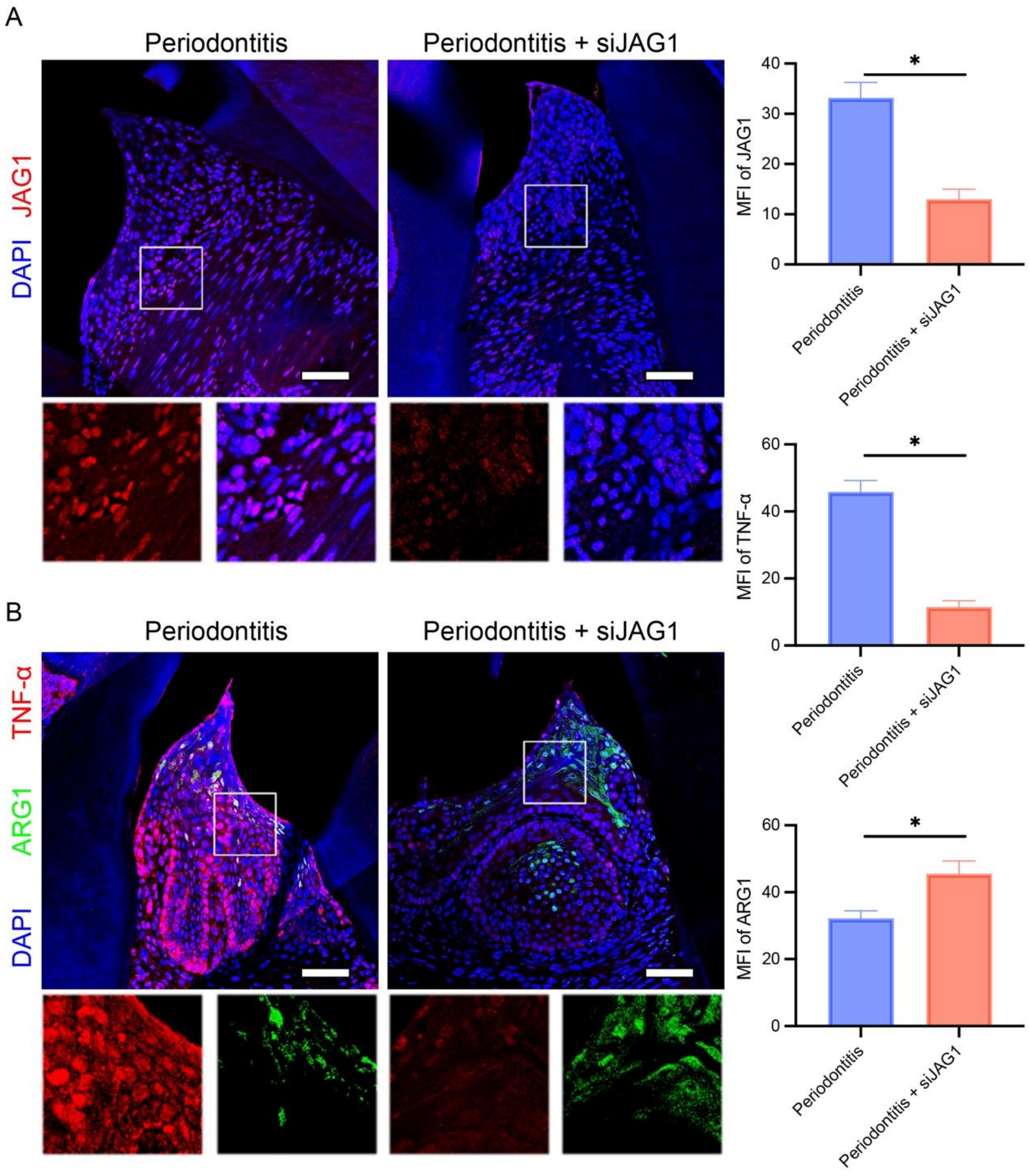

Figure S4. Immunofluorescence staining was performed to examine the gingival tissue of the periodontitis mouse model after siJAG1 treatment. A. Representative images of IF staining showed JAG1 (red) expression in the periodontal tissue of periodontitis model mice treated with saline or siJAG1. B. A. Representative images of IF staining showed TNF-α (red) and ARG1 (green) expression in the periodontal tissue of periodontitis model mice treated with saline or siJAG1. Nuclei were stained with DAPI. Scale bar, 50 mm;
